# RNA sequencing reveals the expression profiles of circRNA and identifies a four-circRNA signature acts as a prognostic marker in esophageal squamous cell carcinoma

**DOI:** 10.1186/s12935-021-01852-9

**Published:** 2021-03-04

**Authors:** Weiwei Wang, Di Zhu, Zhihua Zhao, Miaomiao Sun, Feng Wang, Wencai Li, Jianying Zhang, Guozhong Jiang

**Affiliations:** 1 Department of Pathology, The First Affiliated Hospital of Zhengzhou University, Zhengzhou University, Jian she Dong Road 1, Zhengzhou, 450052 Henan China; 2 Department of Pathology, School of Basic Medicine, Zhengzhou University, Zhengzhou, 450002 China; 3 Henan Key Laboratory for Tumor Pathology, Zhengzhou University, Zhengzhou, 450052 China; 4 Department of Oncology, First Affiliated Hospital of Zhengzhou University, Zhengzhou University, Zhengzhou, 450052 China; 5 State Key Laboratory of Esophageal Cancer Prevention & Treatment, Zhengzhou University, Zhengzhou, 450052 Henan China

**Keywords:** Esophageal squamous cell carcinoma, Prognostic biomarker, circRNA, Signature, Survival

## Abstract

**Background:**

CircRNAs with tissue-specific expression and stable structure may be good tumor prognostic markers. However, the expression of circRNAs in esophageal squamous cell carcinoma (ESCC) remain unknown. We aim to identify prognostic circRNAs and construct a circRNA-related signature in ESCC.

**Methods:**

RNA sequencing was used to test the circRNA expression profiles of 73 paired ESCC tumor and normal tissues after RNase R enrichment. Bioinformatics methods, such as principal component analysis (PCA), t-distributed Stochastic Neighbor Embedding (t-SNE) algorithm, unsupervised clustering and hierarchical clustering were performed to analyze the circRNA expression characteristics. Univariate cox regression analysis, random survival forests-variable hunting (RSFVH), Kaplan–Meier analysis, multivariable Cox regression and ROC (receiver operating characteristic) curve analysis were used to screen the prognostic circRNA signature. Real-time quantitative PCR (qPCR) and fluorescence in situ hybridization(FISH) in 125 ESCC tissues were performed.

**Results:**

Compared with normal tissues, there were 11651 differentially expressed circRNAs in cancer tissues. A total of 1202 circRNAs associated with ESCC prognosis (*P *< 0.05) were identified. Through bioinformatics analysis, we screened a circRNA signature including four circRNAs (hsa_circ_0000005, hsa_circ_0007541, hsa_circ_0008199, hsa_circ_0077536) which can classify the ESCC patients into two groups with significantly different survival (log rank *P *< 0.001), and found its predictive performance was better than that of the TNM stage(0.84 vs. 0.66; 0.65 vs. 0.62). Through qPCR and FISH experiment, we validated the existence of the screened circRNAs and the predictive power of the circRNA signature.

**Conclusion:**

The prognostic four-circRNA signature could be a new prognostic biomarker for ESCC, which has high clinical application value.

## Background

Esophageal squamous cell carcinoma (ESCC) is a malignant epithelial tumor with squamous cell differentiation, accounting for 90% of esophageal cancer. Although the incidence of ESCC shows significant regional differences and is decreasing in recent years, it is still one of the leading causes of cancer deaths [1]. In terms of prognosis, the 5-year survival rate of ESCC is reported to be only 10% [2]. In the past few decades, the progress of ESCC treatment have not significantly increased the life expectancy of patients [3, 4]. The main reason is that most patients are diagnosed at advanced stages without effective treatment, and they are prone to recurrence or metastasis. Therefore, ESCC patients urgently need prognostic markers that can evaluate disease progression and clinical outcome.

Circular RNA, also known as cirRNA, is a class of special non-coding RNA (ncRNA) without a 5′ cap or 3′ tail, consisting mainly of exons and/or introns. Recently, circRNA has been reported to regulate gene expression by competitively binding microRNA and play a key regulatory role in the development of tumors, atherosclerosis, diabetes, and neurological diseases [5]. Due to the special loop structure, circRNA is more resistant to exonuclease and thus has better stability and abundance than linear RNA. Besides, the advantage of circRNA over linear RNA as a prognostic marker is that circRNA can be detected from multiple components such as exosomes, cell-free saliva and plasma [6, 7]. Further, tissue expression specificity of circRNA make it possible as molecular marker [8]. Therefore, circRNA become a research hotspot for prognostic tumor markers.

With the development of biological sequencing technology [9–12], a large number of circRNAs have been discovered, and the role of cirRNAs in human cancer has gradually been revealed. For instance, Wang et al. found that the high expression of circRHOT1 was associated with poor prognosis of hepatocellular carcinoma (HCC), and confirmed that circRHOT1 promoted malignant progression of tumors [13], Liang et al. discovered a new circRNA from breast cancer tissues, named circBMPR2, and identified its role in inhibiting cell proliferation, migration, invasion and tamoxifen resistance by regulating the circBMPR2/miR-553/USP4 axis [14]. A circRNA study of lung cancer found that the oncogene circHIPK3 and linear linHIPK could regulate autophagy, and the circHIPK3/linHIPK3 ratio had potential clinical use as a prognostic factor [15]. The mechanism study of cisplatin (CDDP) treatment resistance in gastric cancer (GC) patients found circAKT3 played an important role and could be a prognostic marker for GC patients receiving CDDP therapy [16]. Research on the pathogenetic and metastatic factor of colon cancer (CC) indicated that circPPP1R12A had a promoting effect and could be a therapeutic target for CC [17]. From the perspective of ESCC, studies on the role of circRNA are increasing. After exploring the circRNA expression profiles of 10 pairs of ESCC tissues by microarray assay, Shi et al. investigated a novel circRNA, termed as hsa_circ_0006168, and confirmed its role in promoting ESCC proliferation, migration and invasion by sponging microRNA-100 and regulating the expression of Mammalian Target of Rapamycin (mTOR) [18]. It was found that Hsa_circ_0000337, hsa_circ_0067934 and ciRS-7 were significantly upregulated in ESCC tissues, and may promote tumor cell proliferation, migration and invasion, suggesting that these circRNAs may become potential therapeutic targets for ESCC [19, 20, 21]. Although circRNA plays an important role in ESCC, there is still a lack of a prognostic circRNA signature based on large samples.

Here, a total of 198 ESCC patients were collected and followed up. We aim to reveal the expression patterns of circRNA in ESCC tissues using RNA sequencing, and to find a clinically valuable circRNA molecular signature that can accurately predict the survival of ESCC patients.

## Materials and methods

### Sample collection and preparation

Anyang is one of the areas with high prevalence of ESCC in China. We collected 73 postoperative patients from Anyang Tumor Hospital with their ESCC and paired non-tumor tissues (approximately 5 cm away from the tumor [22]) and corresponding clinical follow-up data during 2014–2019, then examined the circRNA expression profile of ESCC by next-generation sequencing (NGS) [23]. In addition, we collected an independent validation cohort of 125 ESCC postoperative patients from the same hospital to detect the circRNA expression level using the qRT-PCR. The patients were coded to protect their anonymity. All pathological information of ESCC patients in this study was shown in Additional file 1: Table S1. Tumor-node-metastasis (TNM) classification of the International Union against Cancer (7th edition) was used to categorize. The informed consent document was obtained through the institutional review board. The study was approved by the Ethical Committee of Anyang Tumor Hospital.

### RNA isolation and next generation RNA sequencing analysis

After TRIZOL lysis and purification, total RNA was isolated by the miRNeasy Mini Kit (QIAGEN) with a DNase digestion step. A total amount of 5 μg RNA per sample was used as input material for the RNA sample preparation. First, ribosomal RNA(rRNA) was removed by Epicentre Ribozero™ rRNA Removal Kit (Epicentre, USA), and the rRNA free residue was cleaned up by ethanol precipitation. Subsequently, the linear RNA was digested by 3U RNase R(Epicentre, USA) per μg of RNA. The sequencing libraries were generated by NEBNext Ultra™ Directional RNA Library Prep Kit for Illumina (NEB, USA) following manufacturer’s recommendation. Briefly, fragmentation was carried out using divalent cations under elevated temperature in NEBNext First Strand Synthesis Reaction Buffer(5X). The first strand cDNA was synthesized using random hexamer primers and M-MuLV Reverse Transcriptase (RNaseH-). Then DNA Polymerase I and RNase H were used for second-strand cDNA synthesis. In the dNTPs reaction buffer, dTTP were replaced by dUTP. The remaining overhangs were converted into blunt ends by exonuclease/polymerase activities. After adenylation of 3′ ends of DNA fragments, NEBNext Adaptor with a hairpin loop structure were ligated to prepare for hybridization. In order to select cDNA fragments preferably 250–300 bp in length, the library fragments were purified by AMPure XP system (Beckman Coulter, Beverly, USA). Then, 3 μl USER Enzyme (NEB, USA) was used with size-selected, adaptor-ligated cDNA at 37 °C for 15 min followed by 5 min at 95 °C before PCR. Then PCR was performed with Phusion High-Fidelity DNA polymerase, Universal PCR primers and Index (X) Primer. The product was purified (AMPure XP system), and library quality was assessed on the Agilent Bioanalyzer 2100 system. According to the manufacturer’s instructions, we used TruSeq PE Cluster Kit v3-cBot-HS (Illumia) to cluster the index-coded samples on the cBot Cluster Generation System. After generating the clusters, the libraries were sequenced on an Illumina Hiseq platform and 150 bp paired-end reads were generated. We used bwa to map RNA-Seq reads to hg19, and used the circRNA detection tool CIRI for circRNA identification with default options. Then the alternative splice tool CIRI-AS was used for circRNA internal structure prediction [24]. The data of circBase [25] was combined with the identification results of circRNA. TPM (Transcripts Per Kilobase of exon model per Million mapped reads) was employed to calculate the expression level of individual circRNA. The differential expression of circRNA was assessed by the edgeR algorithm [26, 27].

### Validation of circRNA expression by RT-PCR

CircRNA reverse transcriptions were amplified by TIANScript II RT Kit (KR107, TIANGEN, Beijing, China). We used real-time quantitative PCR (qRT-PCR) to measure the expression of circRNA with TB Green^®^ Premix Ex Taq™ (Tli RNaseH Plus, TaKaRa, Dalian,China). The relative quantification of circRNA expression was normalized by the −ΔΔCt method, and GAPDH was used for normalization with the corresponding primers (Additional file 2: Table S2). All reactions were carried out in triplicate by the StepOnePlus™ Real-Time PCR System (Applied Biosystems) as described previously [28–30]. In order to assess the existence of relevant circRNA candidates, Sanger Sequencing was used to further verify the PCR products at the the circRNA backspliced junction.

### Construction of multi-circRNA prognostic signature

Univariate cox analysis was used to identify circRNAs associated with overall survival (OS). We used the random survival forest algorithm [31] to further screen based on the expression value of circRNA, and then constructed a prognostic risk model. The model was estimated as follows [28, 29, 32].

$$ {\text{Risk Score }}\left( {\text{RS}} \right)\, = \,\sum\limits_{{{\text{i}}\, = \, 1}}^{\text{N}} {({\text{Expression}}_{\text{i}} *{\text{Coefficient}}_{\text{i}} )} $$

N is the number of prognostic circRNA, *Expression*_i_ represents the circRNA expression value, and *Coefficient*_i_ is the Cox regression coefficient of circRNA. We plotted ROC curves and calculated their area under the curve (AUC) values, and then selected the prognostic signature with largest AUC value in the training set [32].

### RNA Fluorescence in situ hybridization (FISH)

FISH probe was designed at the backspliced junction of circRNA and labeled Cy3 fluorescence at 5′ end (Table 1). The esophageal cancer cells were laid into 12-well plates with cell climbing tablets, and FISH hybridization test by RiboTM Fluorescent In Situ Hybridization Kit (RiboBio, Guangzhou, China) was performed after the cells were fully extended. The cells were washed with PBS and fixed with 4% paraformaldehyde for 10 min at room temperature; then 0.5% Triton X-100 (prepared with PBS) was pre-cooled with 1 ml for 5 min at 4 °C; then the pre-hybridization liquid was preheated at 37 °C, and put the pre-hybridization liquid of 200 UL into the cell pore plate, and blocked it at 37 °C for 30 min. Then the probe hybridization liquid was replaced with the prepared one, and the next day the hybridization lasted for 42 °C. The cells were washed by hybrid lotion I (4 * SSC, 0.1% Tween-20), hybrid lotion II (2 * SSC) and hybrid lotion III (1 * SSC) in a constant temperature shaker at 42 °C. After rinsing once with PBS, the nucleic acid dye DAPI was added dropwise and stained for 5 min. After sealing, the cells were observed by a fluorescence microscope (OLMPUS BX51, Japan). All used cell lines including KYSE270, KYSE520, KYSE410, were obtained from German Collection of Microorganisms and Cell Cultures GmbH (DSM: Z https://www.dsmz.de/).

**Table 1:** Identities of circRNAs in the prognostic signature and univariable cox association with prognosis

Database ID	Parent gene	Coefficient	P	Expression with poor prognosis	Chromosome	circRNA type	FISH Probe
hsa_circ_0008199	ATXN10	− 1.181	0.001	Low	chr22:46085591-46136418	Exon	CTGGGTGCTGTTTCTCTTGTCTTGGT
hsa_circ_0007541	USP13	0.742	0.024	High	chr3:179481789-179483636	Intron	CGGCTCAGCAAAATTTCCAGATCCAT
hsa_circ_0000005	CDK11A	0.916	0.004	High	chr1:1586822-1650894	Exon	CATCTTCTTCTCCTCTGTCTTCCATA
hsa_circ_0077536	ATG5	1.065	0.001	High	chr6:106727535-106756366	Exon	CTTGGCAAAAGCAACATTTTGCAATC

FISH: RNA Fluorescence in situ hybridization

### Statistical and bioinformatics analysis

Kaplan–Meier (KM) survival analyses were used to test the difference in survival between groups. Receiver operating characteristic (ROC) curve was performed to calculate the survival prediction power [11]. To explore transcription heterogeneity and to perform initial tissues clustering, we used principal component analysis (PCA) to reduce dimensionality. For the entire dataset, we selected 3 principal components (PCs), which explained more variability than expected by chance using the permutation-based test implemented in Seurat [33]. We used PC loadings as input for a graph-based approach to cluster ESCC samples [34], and as input for t-distributed stochastic neighbor embedding (t-SNE) to simplify it to two dimensions for visualization [35–37]. The R program performed the above analysis, including packages called Seurat, pROC, randomForestSRC and Survival which were downloaded from Bioconductor (http://www.bioconductor.org/). The co-expressed relationships between circRNA and protein-coding genes were computed using Pearson correlation test visualized by Cytoscape. Then Gene Ontology (GO) and the Kyoto Encyclopedia of Genes and Genomes (KEGG) enrichment analyses of the co-expressed genes were performed to predict the biological function by the ClueGo plugin of Cytoscape (version 3.2.3) Functional annotation with P < 0.05 was considered significant.

## Results

### The expression pattern of circRNAs in ESCC

**Fig. 1:**
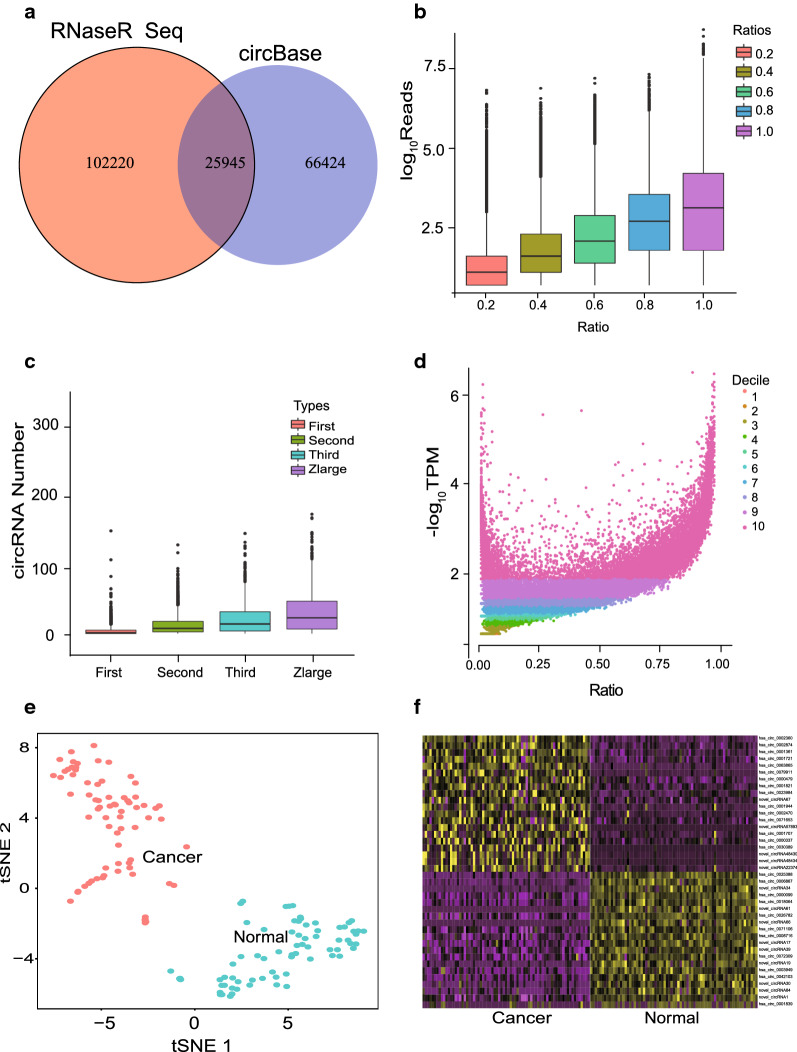
Transcriptomic landscape of circRNAs expression characteristics in ESCC. **a** The overlap of circRNAs in RNA sequencing species by detection tool CIRI and CircBase. **b** Average expression of circRNA abundance (in normalized back splice junction reads) versus average expression of parental expression (in Reads). **c** Distribution of circRNAs detected in the 73 pairs of ESCC transcriptomes. **d** The plot for the mean abundance (adjusted TPM = log10 (TPM + 1)) of every circRNA by the percentage of samples. **e** Two major clusters identified after unsupervised clustering in 146 tissues. Each point represents a single tissue. **f** Heatmap showing top 40 differentially expressed circRNAs between ESCCs and paired normal samples

To identify the expression pattern of circRNA in esophageal squamous cell carcinoma, we extracted total RNA from 73 pairs of tumors and normal tissues adjacent to cancer, and used the RNase R method to enrich the circular transcript. After next-generation sequencing (NGS) analysis, a total of 128,165 circRNAs were detected from these samples, of which 25,945 circRNA candidates were consistent with circBase (Fig. 1a). Then, we analyzed the relationship between the abundance of circRNA and the ratio of circRNA to linear RNA (Fig. 1b). The abundance of circRNA was positively correlated with the ratio of circRNA to linear RNA. When the ratio increased, the abundance of circRNA increased, indicating that highly expressed circRNA accounted for the majority of transcripts transcribed from genes at corresponding positions. The number of circular transcripts increased in proportion to the number of gene exons (Fig. 1c). Moreover, we found thousands of alternative splice in circRNAs, including four types: exon skipping (ES), intron retention (IR), alternative 5′ or 3′ splicing site (A3SS and A5SS) (Fig. 1d). We also found that highly abundant circular transcripts were often extensive and can be detected in most ESCC samples.

### Differentially expressed circRNAs in normal and ESCC tissues

Based on the circRNA expression of 73 pairs of ESCC samples, we performed PCA and t-SNE (See Method) and found the expression of circRNA could be reduced to two distinct expression patterns. According to the circRNA expression pattern, we performed unsupervised clustering on the samples and identified that samples were clustered into two distinct tissues clusters. After checking their clinical information, we found one group of samples were all tumor tissues, while the other group of samples were completely normal tissues (Fig. 1e, Additional file 3: Figure S1). The results showed that the expression of circRNA was different in normal and ESCC tissues. Compared with normal tissues, 11651 circRNAs were differentially expressed in cancer tissues, 5031 were up-regulated, and 6620 were down-regulated. Hierarchical clustering displayed the top 40 differentially expressed circRNAs in the ESCC samples and matched normal samples (Fig. 1f).

**Fig. 2:**
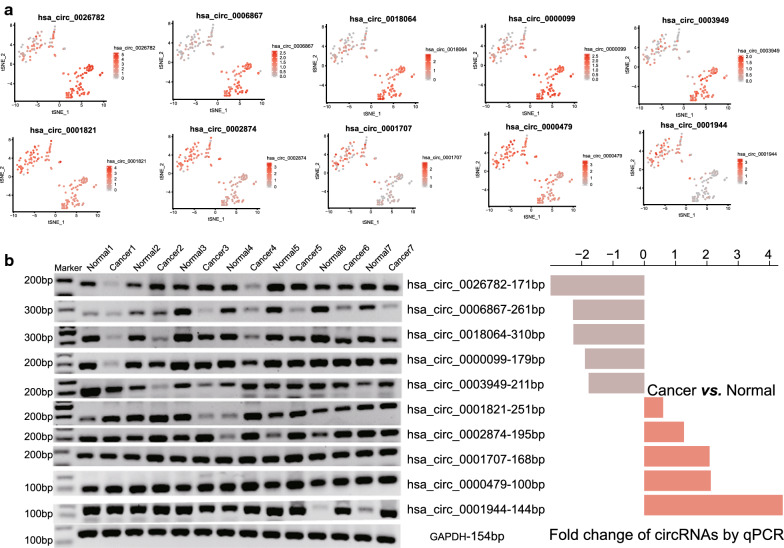
Validation of the selected differentially expressed circRNAs. **a** Visualization of selected circRNAs expression patterns for expression validating. **b** Agarose electrophoresis of selected circRNAs PCR products. **b** Fold change of circRNAs detected by qPCR between Cancer and Normal

To verify the existence of differentially expressed circRNAs in ESCC tissues, we randomly selected 10 differentially expressed circRNAs from the top 20, and conducted qPCR and agarose gel electrophoresis in 7 pairs of tissues randomly chose from the independent ESCC cohort. Figure 2a showed the expression of these ten circRNAs detected in 73 pairs of cancer and adjacent tissues. Compared with normal tissues, five circRNAs were down-regulated in ESCC, while the other five circRNAs were up-regulated. Consistent with the sequencing data, Fig. 2b revealed that all the ten circRNAs and similar expression pattern can be detected in seven paired ESCC and normal tissues (the primers showed in Additional file 4: Figure S2), indicating that circRNA was stable and can be further used as a prognostic marker.

### Identification of survival-related circRNAs and construction of a prognostic circRNA signature in ESCC

**Fig. 3:**
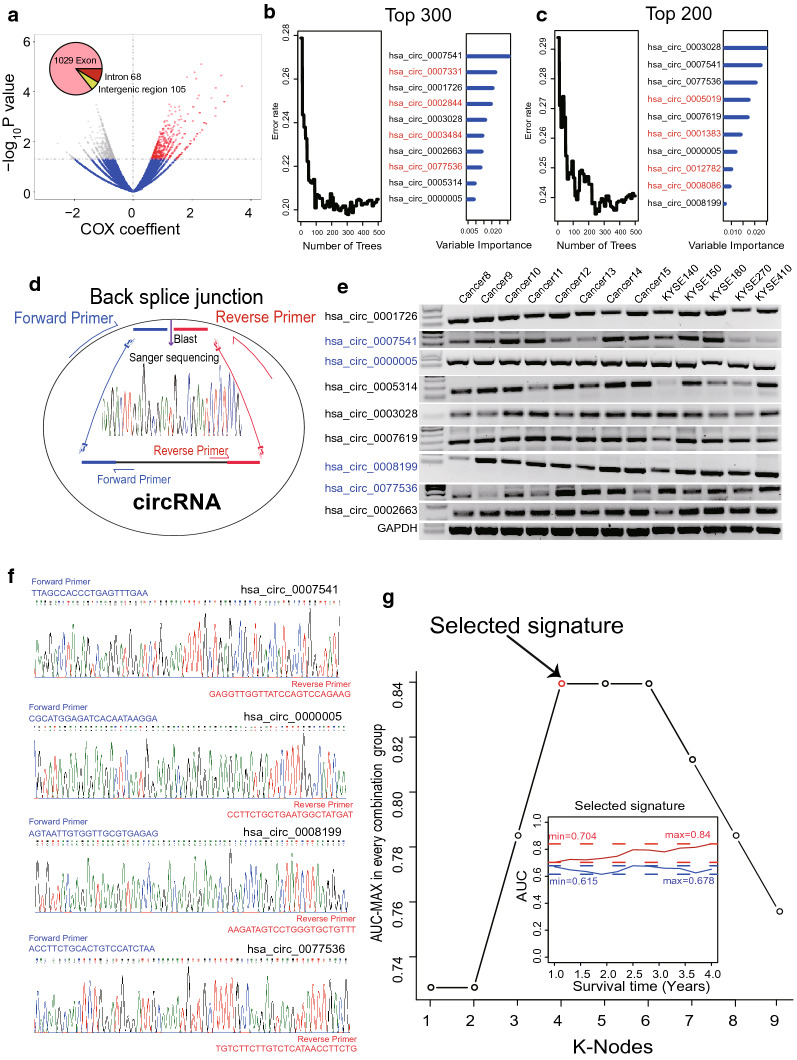
Constructing the prognostic circRNA model in the RNA-seq dataset. **a** Univariate Cox analysis of the circRNAs expression with prognosis in RNA-seq dataset. The error rate for the data in random survival forests analysis as a function of trees and according to important score to filter Top 300 (**b**) or 200 (**c**) high expression of prognostic circRNAs. **d** Flow chart for identification of existing circRNA. **e** Agarose electrophoresis of selected circRNAs PCR products. **f** The results of Sanger sequence for the four circRNAs in the prognostic signature. **g** Screening out the multi-circRNA signature with largest AUC from all 512 signatures which were calculated by ROC for k = 1, 2…… 9 in the plot

Based on the expression data and the corresponding clinical follow-up information of 73 ESCC samples, we performed univariate cox analysis and identified 1202 circRNAs that were significantly associated with ESCC OS (*P *< 0.05). Among them, 1029 circRNAs were from exons, 68 circRNAs were from intergenic regions and 105 were from introns (Additional file 5: Table S3, Fig. 3a).

Considering that genes as prognostic markers should be highly expressed in tissues, we first ranked the expression of the 1202 circRNAs, and then performed two times random survival forests-variable hunting (RSFVH) analyses on the 300 or 200 circRNAs with the highest expression. Through discarding one-third of the least important circRNAs in each step according to the importance score, ten circRNAs were screened in two analyses, 3 circRNAs of which were the same (Fig. 3b and c). Therefore, a total of 17 highly expressed circRNAs were screened out, which are related to the prognosis of ESCC. According to the sequence in Circbase, we designed 17 pairs of reverse primers for the above 17 circRNAs. After PCR amplification, target band recovery and Sanger sequencing, we blasted the sequence at the the back-spliced junction of each circRNA (Fig. 3d) to verify the expression of 17 circRNAs in ESCC tissues and cell lines. As a result, we found that 9 circRNAs were actually expressed(Fig. 3e, f, Additional file 6: Figure S3), while the other 8 circRNAs can not be detected in ESCC.

To construct a prognostic signature with good performance from 2^9^−1 = 511 combinations, we performed ROC analyses in the dataset with 73 ESCC samples which was considered as the training dataset (Additional file 7: Table S4). The circRNA combination composed of hsa_circ_0005314, hsa_circ_0007541, hsa_circ_0000005 and hsa_circ_0077536 was selected since its AUC value was the largest (Fig. 3g, Table 1). The risk score of the selected circRNA signature was as follows:Risk score = (− 1.181 × expression value of hsa_circ_0008199) + (0.742 × expression value of hsa_circ_0007541) + (0.916 × expression value of hsa_circ_0000005) + (1.065 × expression value of hsa_circ_0077536). The AUC of the screened circRNA signature in the prognostic model was 0.839, demonstrating its good performance in survival prediction. Interestingly, subcellular localization experiment showed that all four circRNAs were mainly located in the nucleus (Additional file 8: Figure S4).

### Survival prediction ability of the circRNA signature

**Fig. 4:**
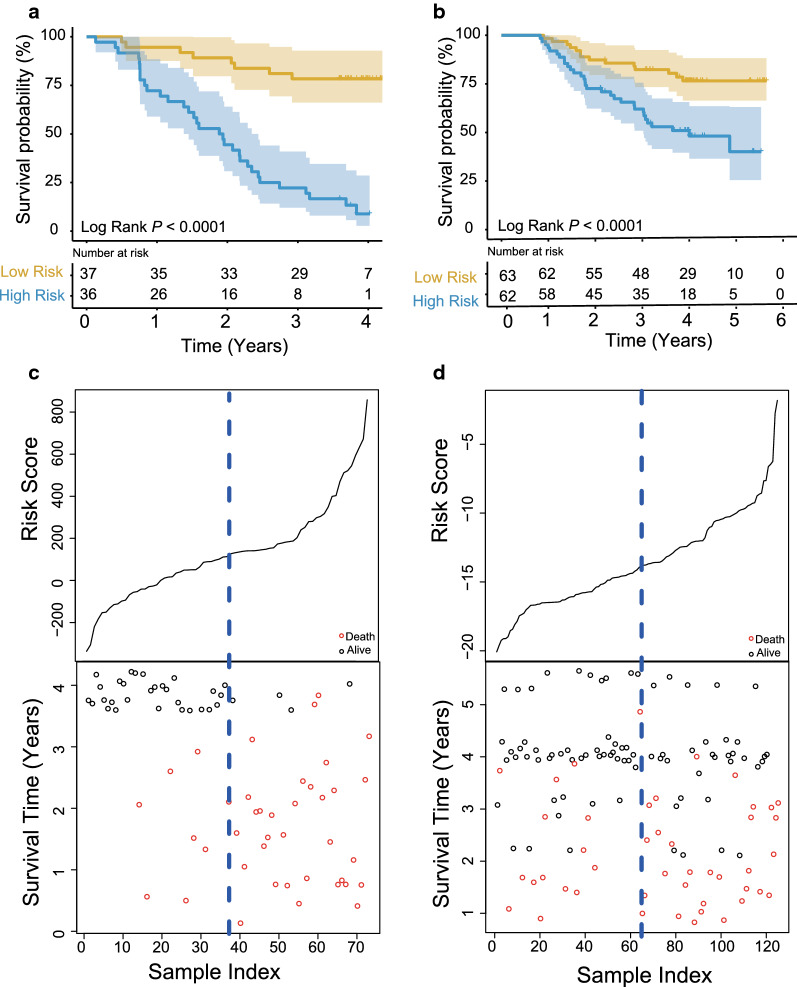
The circRNA signature classification power for ESCC prognosis. Kaplan–Meier curves classify ESCCs into two different risk groups based on the risk score of the signature in the training (**a**) and test (**b**) dataset. Risk score distribution, survival status of ESCC patients in high- and low-risk groups by the four-circRNA signature in the two datasets (**c**, **d**)

After obtaining the risk score of every ESCC patient in the training group, patients were classified into a high-risk (*n *= 36) group and a low-risk (*n *= 37) group based on the median risk score. KM analysis found that the prognosis of the two groups was significantly different. The OS of high-risk ESCC patients was significantly shorter than that of the low-risk group (median survival: 1.91 years vs. 3.72 years, log-rank test *P *< 0.001; Fig. 4a). The 3-year survival rate of the high-risk group was only 19.4%, while that of the low-risk group was 78.4%. To varify the prognostic performance, the circRNA signature was also evaluated in another independent ESCC dataset (*n *= 125). We performed qPCR experiment to test the expression of four circRNAs and calculated risk scores and median risk score based on the circRNA signature for 125 ESCC samples. The prognosis of patients in the high-risk and low-risk groups in the test group was significantly different, as shown in Fig. 4b (log-rank test *P *< 0.001). The 3-year survival rate of ESCC patients in the low-risk group was still significantly higher than that of the high-risk group.

From Fig. 4c, d, we can see the relationship between survival time and risk score in the training and test datasets. ESCC patients with low risk scores survived longer, while patients with high scores survive shorter.

### The four-circRNA signature is an independent prognostic marker for ESCC

We explored the relationship between circRNA signature and clinicopathological factors, including age, sex, smoking, drinking, T stage, N stage, and pTNM stage, and found that the TNM stage in the training group was related to the signature (Additional file 9: Table S5). Therefore, whether the circRNA signature is an independent factor for ESCC survival is necessary to confirm. Using the risk score based on the circRNA signature and other clinical features as variables, we performed multivariable Cox regression analysis in the training and test datasets. The results showed the circRNA signature for ESCC survival prediction was indeed independent of other clinical features (High-risk group vs. Low-risk group, HR training = 2.79, 95% CI 1.484–5.260, *P *= 0.001, *n *= 73; HR test = 2.58, 95% CI 1.363–4.896, *P *= 0.004, *n *= 125, Table 2).

**Table 2:** Univariable and multivariable Cox regression analysis of the circRNA signature and survival of ESCCs in the training and test group

		Univariable analysis				Multivariable analysis			
Variables		HR	95% CI of HR		*P*	HR	95% CI of HR		*P*
			Lower	Upper			Lower	Upper	
Training dataset (*n *= 73)									
Sex	Male vs. Female	1.375	0.718	2.635	0.337	1.336	0.682	2.617	0.398
Age	> 62 vs. ≤ 62	1.241	0.667	2.311	0.495	1.585	0.819	3.066	0.171
pTNM stage	III, IV vs. I, II	2.424	1.362	4.314	0.003	1.639	0.884	3.039	0.117
circRNA-signature	High risk vs. low risk	7.746	3.519	17.051	0.000	6.705	2.951	15.237	0.000
Test set (*n *= 125)									
Sex	Male vs. Female	1.185	0.659	2.130	0.570	1.109	0.614	2.003	0.732
Age	> 62 vs. ≤ 62	0.987	0.543	1.793	0.966	1.084	0.596	1.974	0.791
pTNM stage	III, IV vs. I, II	1.825	1.163	2.863	0.009	1.635	1.032	2.590	0.036
circRNA signature	High risk vs. low risk	2.794	1.484	5.260	0.001	2.583	1.363	4.896	0.004

### Comparing the survival predictive power of the four-circRNA signature and TNM stage

**Fig. 5:**
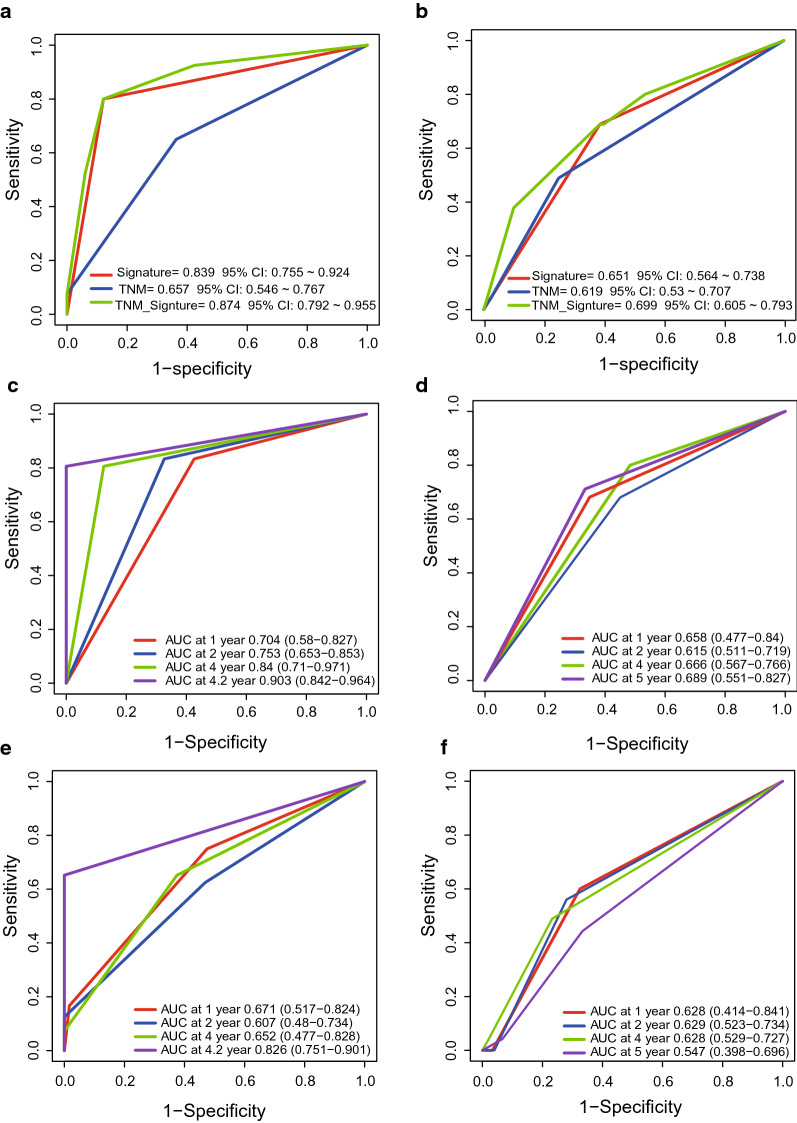
Comparing the survival prediction power of the signature with TNM stage by ROC in the training and test datasets (**a**, **b**). TimeROC analysis of survival prediction power for the signature (**c**, **d**) and TNM stage (**e**, **f**) in the two datasets

TNM stage is a commonly used prognostic indicator in clinical practice. Therefore, we performed ROC analysis to compare the predictive performance of the four-circRNA signature with TNM stage [38, 39]. In the training and test datasets (n = 73/125), the area under the ROC curve (AUC) of the circRNA signature was significantly larger than that of TNM stage (AUC training = 0.839 vs. 0.657; AUC test = 0.651 vs. 0.619, Fig. 5a, b). When the circRNA signature was used in combination with the TNM stage in both training and test datasets, the AUC value was greater than the TNM stage or the circRNA signature alone (AUC = 0.874/0.699, 95% CI = 0.792–0.955/0.605–0.793, Fig. 5a, b).

To further test the good predictive ability of the signature, we conducted time ROC analysis in the two ESCC groups. The AUC of the signature was 0.704/0.753/0.903 at 1/2/4.2 years in the training set and 0.658/0.615/0.689 at 1/2/5 years in the test set (Fig. 5c, d), while the AUC of TNM was 0.671/0.607/0.826 at 1/2/4.2 years in the training set and 0.628/0.629/0.547 at 1/2/5 years, indicating the four-circRNA signature outperformed TNM stage in terms of ESCC prognosis.

### Functional prediction of circRNAs in the signature

**Fig. 6:**
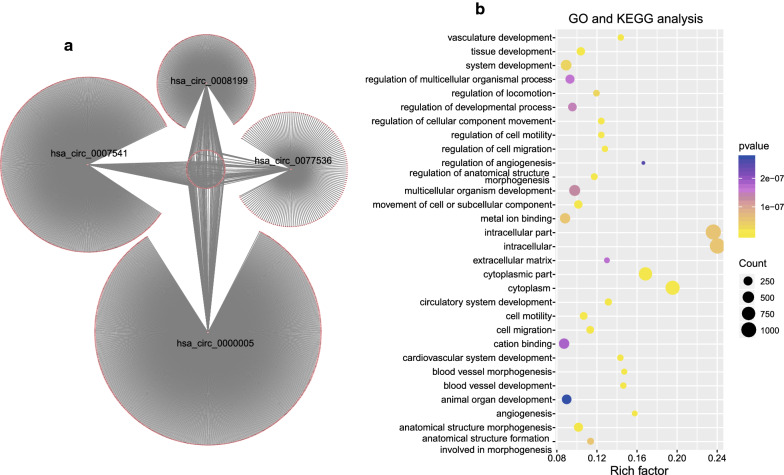
Co-expression network (**a**) and function analysis (**b**) of the circRNAs in the signature

Through Pearson correlation test in the 73 ESCC expression profiles (|Pearson coefficient| > 0.3, P < 0.05, Fig. 6a), co-expression network of the four circRNAs and the 1425 protein-coding genes was constructed. GO and KEGG analysis suggested the four circRNAs were significantly enriched in 78 different GO terms and KEGG pathways (P < 0.05), which implies that four circRNAs may affect important biological processes, such as circulatory system development, angiogenesis and cell migration (Fig. 6b).

## Discussion

Esophageal squamous cell carcinoma has a poor prognosis, but lacks good prognostic markers. Recently, it has been continuously reported that circRNA is involved in the occurrence and progression of tumors [19, 20, 40, 41]. However, the expression characteristics and roles of circRNAs in ESCC are still elusive. Thus, we sequenced 73 pairs of esophageal squamous cell carcinoma and adjacent normal tissues to reveal the circRNA expression profile, and constructed a prognostic circRNA signature.

CircRNAs are a class of closed continuous loop non-coding RNA molecules. Due to its various biological functions, including acting as miRNA sponge to regulate the expression of downstream target genes, regulating gene transcription and translating proteins [42–46], circRNA has attracted the attention of scientists for its potential role in ESCC. As mentioned in the background, some studies have carried out circRNA sequencing on ESCC tissues or cell lines. It is reported that certain circRNAs can promote cell proliferation, migration, and invasion of ESCC, such as hsa_circ_0006168, hsa_circ_0000337, hsa_circ_0067934 [18–20]. However, there is little research based on clinical large cohorts of ESCC and high-throughput circRNA sequencing. Our study included 198 ESCC patients from high-risk areas in China, of which 73 patients recieved circRNA sequencing and the other 125 patients were used to verify the expression of circRNA. We found that the abundance of circRNA in cancer tissues was lower than that in normal tissues, 5031 up-regulated and 6620 down-regulated.

CircRNAs fall into three broad categories based on their source in the genome: exonic, intronic and intergenic [17, 47]. The circRNAs from gene exons are the most common [47, 48]. Our sequencing results showed that most of the circRNAs expressed in ESCC were mainly derived from exons. Bioinformatics analysis revealed differences in circRNA expression patterns between tumor and normal tissues (Additional file 10: Table S6), suggesting ESCC tissues could be distinguished by the expression of circRNA. Further analysis of the expression profile of esophageal squamous cell carcinoma found that 17 circRNAs were significantly associated with OS, and a four-circRNA signature was constructed for ESCC, which had a good survival predictive performance in the training dataset and another independent cohort of 125 ESCC patients. The independence of the predictive power of circRNA signature was also confirmed. Thus, we suggest that the four-circRNA signature is a potential prognostic marker for patients with ESCC.

TNM stage is a commonly used tumor classification standard in clinical practice and a recognized prognostic marker [49, 50]. However, TNM stage is flawed in prognostic assessment. We found that the prognostic ability of signature is better than TNM stage, suggesting that the strong prognostic ability of the four-circRNA signature. Consistent with the findings of some scholars, the combination of TNM classification and molecular marker can more accurately predict outcome of ESCC patients [51], indicating the signature is useful for prognosis evaluation.

In the process of exploring circRNA-based prognostic signature, we discovered some new circRNAs. The 10 differentially expressed circRNAs in tumor tissues and adjacent tissues (hsa_circ_0026782, hsa_circ_0006867, hsa_circ_0018064, hsa_circ_0000099, hsa_circ_0003949, hsa_circ_0001821, hsa_circ_0002874, hsa_circ_0001707, hsa_circ_0000479, hsa_circ_0001944)) were amplified by qPCR in ESCC tissues. Agarose gel electrophoresis verified the expression of 9 prognostic cicRNAs (hsa_circ_0001726, hsa_circ_0007541, hsa_circ_0000005, hsa_circ_0005314, hsa_circ_0003028, hsa_circ_0007619, hsa_circ_0008199, hsa_circ_0077536, hsa_circ_0002663). Most importantly, we detected the expression of the four circRNAs of the signature in ESCC tissues and cell lines by agarose gel electrophoresis, sanger sequencing and FISH. This study has confirmed the existence of the four circRNAs and their prognostic significance. Coincidentally, we found all four circRNAs (hsa_circ_0005314, hsa_circ_0007541, hsa_circ_0000005 and hsa_circ_0077536) were mainly expressed in the nucleus. Therefore, we speculate that these cirRNAs may interact with some proteins on the nucleus to regulate the development of ESCC. What are the specific functions of these circRNAs and the mechanisms by which they regulate the development of ESCC remain to be further studied.

The high tissue specificity, high expression abundance [52], high stability [53] and other properties of the circRNA contribute to the clinical application of circRNA signature. Furthermore, circRNAs are abundantly enriched in exosomes. This means that they are widely present in body fluids, including blood, tears, urine, saliva, milk, ascites, etc., and are easily detected, which increases the clinical value of using four-circRNA signature to analyze the prognosis of ESCC patients. From a pan-cancer dataset, circRNAs in body fluids have been discovered to be novel biomarkers to monitor cancer development and progression [54]. For ESCC, researchers have detected that cricRNAs in plasma have prognostic value, such as circ-TTC17 [55], hsa_circ_0004771 [56] and circ-SLC7A5 [57]. The prognostic value of the four circRNAs from the signature in body fluids has not been reported, and we plan to explore it in future studies.

In the analysis and validation of the circRNA expression profile of ESCC patients, we found that not all circRNAs identified by database or software are expressed in ESCC tissues and cells. This is worthy of the attention of the researchers, reminding researchers that the circRNA molecules for bioinformatics mining need to be experimentally verified. Otherwise, false positives may mislead the research direction.

## Conclusion

In summary, we investigated the expression of circRNAs in ESCC and identified a prognostic signature that could divide patients into groups with different survival. As far as we know, it is the first circRNA signature that can predict the overall survival of ESCC patients with high prediction accuracy.

## Supplementary Information

**Additional file 1: Table S1.** Summary of patient demographics and clinical characteristics.

**Additional file 2: Table S2.** The detail of circRNAs and GAPDH primers in this study.

**Additional file 3: Figure S1.** ESCC samples were clustered into two distinct tissues group by circRNA.

**Additional file 4: Figure S2.** Sanger sequencing results of the ten differentially expressed circRNAs.

**Additional file 5:** Table S3: circRNAs of Univariate Cox regression analysis (P < 0.05) in the training set (n = 73).

**Additional file 6: Figure S3.** Sanger sequencing results of the remaining 5 prognostic circRNAs except the 4 circRNAs in the signature.

**Additional file 7: Table S4.** The 511 signatures comprising different circRNAs in the training dataset (n = 73).

**Additional file 8: Figure S4.** The subcellular localization experiment showed that all four circRNAs were located in the nucleus.

**Additional file 9: Table S5.** Association of the circRNA signature with clinicopathological characteristics in ESCC patients.

**Additional file 10: Table S6:** Differentially regulated circRNA analysis between ESCC versus normal.

## Data Availability

The authors declare that the data are available if necessary.

## Abbreviations

ESCC: Esophageal squamous cell carcinoma
PCA: Principal component analysis
t-SNE: t-distributed Stochastic Neighbor Embedding
qPCR: Real-time quantitative PCR
FISH: Fluorescence in situ hybridization
RSFVH: Random survival forests-variable hunting
ROC: Receiver operating characteristic
KM: Kaplan–Meier
AUC: Area under the ROC curve
CI: Confidence interval
OS: Overall survival

## References

[1] Bray F, Ferlay J, Soerjomataram I, Siegel RL, Torre LA, Jemal A (2018). Global cancer statistics 2018: GLOBOCAN estimates of incidence and mortality worldwide for 36 cancers in 185 countries. Cancer J Clin.

[2] Centers for Disease C, Prevention (1993). Mortality trends for selected smoking-related cancers and breast cancer–United States, 1950–1990. Morbid Mortal Wkly..

[3] Zeng H, Zheng R, Zhang S, Zuo T, Xia C, Zou X, Chen W (2016). Esophageal cancer statistics in China, 2011: estimates based on 177 cancer registries. Thorac Cancer.

[4] Chen W, Zheng R, Baade PD, Zhang S, Zeng H, Bray F, Jemal A, Yu XQ, He J (2016). Cancer statistics in China, 2015. Cancer J Clin.

[5] Kristensen LS, Andersen MS, Stagsted LVW, Ebbesen KK, Hansen TB, Kjems J (2019). The biogenesis, biology and characterization of circular RNAs. Nat Rev Genet.

[6] Li Y, Zheng Q, Bao C, Li S, Guo W, Zhao J, Chen D, Gu J, He X, Huang S (2015). Circular RNA is enriched and stable in exosomes: a promising biomarker for cancer diagnosis. Cell Res.

[7] Bahn JH, Zhang Q, Li F, Chan TM, Lin X, Kim Y, Wong DT, Xiao X (2015). The landscape of microRNA, Piwi-interacting RNA, and circular RNA in human saliva. Clin Chem.

[8] Meng S, Zhou H, Feng Z, Xu Z, Tang Y, Li P, Wu M (2017). CircRNA: functions and properties of a novel potential biomarker for cancer. Mol Cancer.

[9] Tripathy D, Harnden K, Blackwell K, Robson M (2014). Next generation sequencing and tumor mutation profiling: are we ready for routine use in the oncology clinic?. BMC Med.

[10] Biesecker LG, Burke W, Kohane I, Plon SE, Zimmern R (2012). Next-generation sequencing in the clinic: are we ready?. Nat Rev Genet.

[11] Guo JC, Fang SS, Wu Y, Zhang JH, Chen Y, Liu J, Wu B, Wu JR, Li EM, Xu LY (2019). CNIT: a fast and accurate web tool for identifying protein-coding and long non-coding transcripts based on intrinsic sequence composition. Nucleic Acids Res.

[12] Wu Y, Zhang F, Yang K, Fang S, Bu D, Li H, Sun L, Hu H, Gao K, Wang W (2019). SymMap: an integrative database of traditional Chinese medicine enhanced by symptom mapping. Nucleic Acids Res.

[13] Wang L, Long H, Zheng Q, Bo X, Xiao X, Li B (2019). Circular RNA circRHOT1 promotes hepatocellular carcinoma progression by initiation of NR2F6 expression. Mol Cancer.

[14] Liang Y, Song X, Li Y, Ma T, Su P, Guo R, Chen B, Zhang H, Sang Y, Liu Y (2019). Targeting the circBMPR2/miR-553/USP4 axis as a potent therapeutic approach for breast cancer. Mol Ther Nucleic Acids.

[15] Chen X, Mao R, Su W, Yang X, Geng Q, Guo C, Wang Z, Wang J, Kresty LA, Beer DG (2019). Circular RNA circHIPK3 modulates autophagy via MIR124-3p-STAT3-PRKAA/AMPKalpha signaling in STK11 mutant lung cancer. Autophagy.

[16] Huang X, Li Z, Zhang Q, Wang W, Li B, Wang L, Xu Z, Zeng A, Zhang X, Zhang X (2019). Circular RNA AKT3 upregulates PIK3R1 to enhance cisplatin resistance in gastric cancer via miR-198 suppression. Mol Cancer.

[17] Zheng X, Chen L, Zhou Y, Wang Q, Zheng Z, Xu B, Wu C, Zhou Q, Hu W, Wu C (2019). A novel protein encoded by a circular RNA circPPP1R12A promotes tumor pathogenesis and metastasis of colon cancer via Hippo-YAP signaling. Mol Cancer.

[18] Shi Y, Guo Z, Fang N, Jiang W, Fan Y, He Y, Ma Z, Chen Y (2019). hsa_circ_0006168 sponges miR-100 and regulates mTOR to promote the proliferation, migration and invasion of esophageal squamous cell carcinoma. Biomed Pharmacother.

[19] Song H, Xu D, Shi P, He B, Li Z, Ji Y, Agbeko CK, Wang J (2019). Upregulated circ RNA hsa_circ_0000337 promotes cell proliferation, migration, and invasion of esophageal squamous cell carcinoma. Cancer Manag Res.

[20] Xia W, Qiu M, Chen R, Wang S, Leng X, Wang J, Xu Y, Hu J, Dong G, Xu PL (2016). Circular RNA has_circ_0067934 is upregulated in esophageal squamous cell carcinoma and promoted proliferation. Sci Rep.

[21] Huang H, Wei L, Qin T, Yang N, Li Z, Xu Z (2019). Circular RNA ciRS-7 triggers the migration and invasion of esophageal squamous cell carcinoma via miR-7/KLF4 and NF-kappaB signals. Cancer Biol Ther.

[22] Tang L, Liou YL, Wan ZR, Tang J, Zhou Y, Zhuang W, Wang G (2019). Aberrant DNA methylation of PAX1, SOX1 and ZNF582 genes as potential biomarkers for esophageal squamous cell carcinoma. Biomed Pharmacother.

[23] Cao HH, Zhang SY, Shen JH, Wu ZY, Wu JY, Wang SH, Li EM, Xu LY (2015). A three-protein signature and clinical outcome in esophageal squamous cell carcinoma. Oncotarget.

[24] Gao Y, Wang J, Zheng Y, Zhang J, Chen S, Zhao F (2016). Comprehensive identification of internal structure and alternative splicing events in circular RNAs. Nat Commun.

[25] Glazar P, Papavasileiou P, Rajewsky N (2014). circBase: a database for circular RNAs. RNA.

[26] Robinson MD, McCarthy DJ, Smyth GK (2010). edgeR: a Bioconductor package for differential expression analysis of digital gene expression data. Bioinformatics.

[27] Varet H, Brillet-Gueguen L, Coppee JY, Dillies MA (2016). SARTools: a DESeq2- and EdgeR-Based R pipeline for comprehensive differential analysis of RNA-Seq data. PLoS ONE.

[28] Guo JC, Xie YM, Ran LQ, Cao HH, Sun C, Wu JY, Wu ZY, Liao LD, Zhao WJ, Fang WK (2017). L1CAM drives oncogenicity in esophageal squamous cell carcinoma by stimulation of ezrin transcription. J Mol Med.

[29] Guo JC, Li CQ, Wang QY, Zhao JM, Ding JY, Li EM, Xu LY (2016). Protein-coding genes combined with long non-coding RNAs predict prognosis in esophageal squamous cell carcinoma patients as a novel clinical multi-dimensional signature. Mol BioSyst.

[30] Zhang XD, Huang GW, Xie YH, He JZ, Guo JC, Xu XE, Liao LD, Xie YM, Song YM, Li EM (2018). The interaction of lncRNA EZR-AS1 with SMYD3 maintains overexpression of EZR in ESCC cells. Nucleic Acids Res.

[31] Li J, Chen Z, Tian L, Zhou C, He MY, Gao Y, Wang S, Zhou F, Shi S, Feng X (2014). LncRNA profile study reveals a three-lncRNA signature associated with the survival of patients with oesophageal squamous cell carcinoma. Gut.

[32] Guo JC, Wu Y, Chen Y, Pan F, Wu ZY, Zhang JS, Wu JY, Xu XE, Zhao JM, Li EM (2018). Protein-coding genes combined with long noncoding RNA as a novel transcriptome molecular staging model to predict the survival of patients with esophageal squamous cell carcinoma. Cancer Commun.

[33] Macosko EZ, Basu A, Satija R, Nemesh J, Shekhar K, Goldman M, Tirosh I, Bialas AR, Kamitaki N, Martersteck EM (2015). Highly parallel genome-wide expression profiling of individual cells using nanoliter droplets. Cell.

[34] Villani AC, Satija R, Reynolds G, Sarkizova S, Shekhar K, Fletcher J, Griesbeck M, Butler A, Zheng S, Lazo S (2017). Single-cell RNA-seq reveals new types of human blood dendritic cells, monocytes, and progenitors. Science.

[35] Jamieson AR, Giger ML, Drukker K, Li H, Yuan Y, Bhooshan N (2010). Exploring nonlinear feature space dimension reduction and data representation in breast Cadx with Laplacian eigenmaps and t-SNE. Med Phys.

[36] Li W, Cerise JE, Yang Y, Han H (2017). Application of t-SNE to human genetic data. J Bioinform Comput Biol.

[37] Taskesen E, Reinders MJ (2016). 2D representation of transcriptomes by t-SNE exposes relatedness between human tissues. PLoS ONE.

[38] Tse LA, Dai J, Chen M, Liu Y, Zhang H, Wong TW, Leung CC, Kromhout H, Meijer E, Liu S (2015). Prediction models and risk assessment for silicosis using a retrospective cohort study among workers exposed to silica in China. Sci Rep.

[39] Heagerty PJ, Lumley T, Pepe MS (2000). Time-dependent ROC curves for censored survival data and a diagnostic marker. Biometrics.

[40] He J, Xie Q, Xu H, Li J, Li Y (2017). Circular RNAs and cancer. Cancer Lett.

[41] Memczak S, Jens M, Elefsinioti A, Torti F, Krueger J, Rybak A, Maier L, Mackowiak SD, Gregersen LH, Munschauer M (2013). Circular RNAs are a large class of animal RNAs with regulatory potency. Nature.

[42] Han D, Li J, Wang H, Su X, Hou J, Gu Y, Qian C, Lin Y, Liu X, Huang M (2017). Circular RNA circMTO1 acts as the sponge of microRNA-9 to suppress hepatocellular carcinoma progression. Hepatology.

[43] Hansen TB, Jensen TI, Clausen BH, Bramsen JB, Finsen B, Damgaard CK, Kjems J (2013). Natural RNA circles function as efficient microRNA sponges. Nature.

[44] Du WW, Yang W, Li X, Awan FM, Yang Z, Fang L, Lyu J, Li F, Peng C, Krylov SN (2018). A circular RNA circ-DNMT1 enhances breast cancer progression by activating autophagy. Oncogene.

[45] Zhong Y, Du Y, Yang X, Mo Y, Fan C, Xiong F, Ren D, Ye X, Li C, Wang Y (2018). Circular RNAs function as ceRNAs to regulate and control human cancer progression. Mol Cancer.

[46] Dong Y, He D, Peng Z, Peng W, Shi W, Wang J, Li B, Zhang C, Duan C (2017). Circular RNAs in cancer: an emerging key player. J Hematol Oncol.

[47] Vicens Q, Westhof E (2014). Biogenesis of circular RNAs. Cell.

[48] Chen I, Chen CY, Chuang TJ (2015). Biogenesis, identification, and function of exonic circular RNAs. Wiley Interdiscipl reviews RNA.

[49] Sobin LH, Fleming ID, TNM Classification of Malignant Tumors, fifth edition (1997). Union Internationale Contre le Cancer and the American Joint Committee on Cancer. Cancer.

[50] Sobin LH, Hermanek P, Hutter RV (1988). TNM classification of malignant tumors. A comparison between the new (1987) and the old editions. Cancer.

[51] Takeno S, Noguchi T, Takahashi Y, Fumoto S, Shibata T, Kawahara K (2007). Assessment of clinical outcome in patients with esophageal squamous cell carcinoma using TNM classification score and molecular biological classification. Ann Surg Oncol.

[52] Jeck WR, Sorrentino JA, Wang K, Slevin MK, Burd CE, Liu J, Marzluff WF, Sharpless NE (2013). Circular RNAs are abundant, conserved, and associated with ALU repeats. RNA.

[53] Chen LL (2016). The biogenesis and emerging roles of circular RNAs. Nat Rev Mol Cell Biol.

[54] Wang S, Zhang K, Tan S, Xin J, Yuan Q, Xu H, Xu X, Liang Q, Christiani DC, Wang M (2021). Circular RNAs in body fluids as cancer biomarkers: the new frontier of liquid biopsies. Mol Cancer.

[55] Wang Q, Zhang Q, Sun H, Tang W, Yang L, Xu Z, Liu Z, Jin H, Cao X (2019). Circ-TTC17 promotes proliferation and migration of esophageal squamous cell carcinoma. Dig Dis Sci.

[56] Huang E, Fu J, Yu Q, Xie P, Yang Z, Ji H, Wang L, Luo G, Zhang Y, Li K (2020). CircRNA hsa_circ_0004771 promotes esophageal squamous cell cancer progression via miR-339-5p/CDC25A axis. Epigenomics.

[57] Wang Q, Liu H, Liu Z, Yang L, Zhou J, Cao X, Sun H (2020). Circ-SLC7A5, a potential prognostic circulating biomarker for detection of ESCC. Cancer Genet.

