# Supplementary material for: RNA sequencing reveals the expression profiles of circRNA and identifies a four-circRNA signature acts as a prognostic marker in esophageal squamous cell carcinoma
Source: Cancer Cell Int. 2021 Mar 4;21:151. doi: 10.1186/s12935-021-01852-9 (PMC7934454; doi:10.1186/s12935-021-01852-9)
Supplement: Supplementary file 1 — Additional file 1: Table S1. Summary of patient demographics and clinical characteristics. [file 12935_2021_1852_MOESM1_ESM.doc]

| **Table S1. Summary of patient demographics and clinical characteristics.** | | |
| --- | --- | --- |
| **Characteristic** | **Sequenced set** | **Validation set** |
| **Age (years)** |  |  |
| > 62 | 41 | 50 |
| ≤ 62 | 32 | 75 |
| **Sex** |  |  |
| female | 23 | 69 |
| Male | 50 | 56 |
| **Vital status** |  |  |
| Living | 33 | 45 |
| Dead | 40 | 80 |
| **T stage** |  |  |
| T1 | 0 | 8 |
| T2 | 18 | 31 |
| T3 | 54 | 85 |
| T4 | 1 | 1 |
| **N stage** |  |  |
| N0 | 35 | 80 |
| N1 | 22 | 34 |
| N2 | 13 | 8 |
| N3 | 3 | 3 |
| **pTNM stage** |  |  |
| Stage Ⅰ | 0 | 0 |
| Stage Ⅱ | 35 | 83 |
| Stage Ⅲ | 35 | 38 |
| Stage Ⅳ | 3 | 4 |
