# Supplementary figures and images for: RNA sequencing reveals the expression profiles of circRNA and identifies a four-circRNA signature acts as a prognostic marker in esophageal squamous cell carcinoma
Source: Cancer Cell Int. 2021 Mar 4;21:151. doi: 10.1186/s12935-021-01852-9 (PMC7934454; doi:10.1186/s12935-021-01852-9)

Figure S1

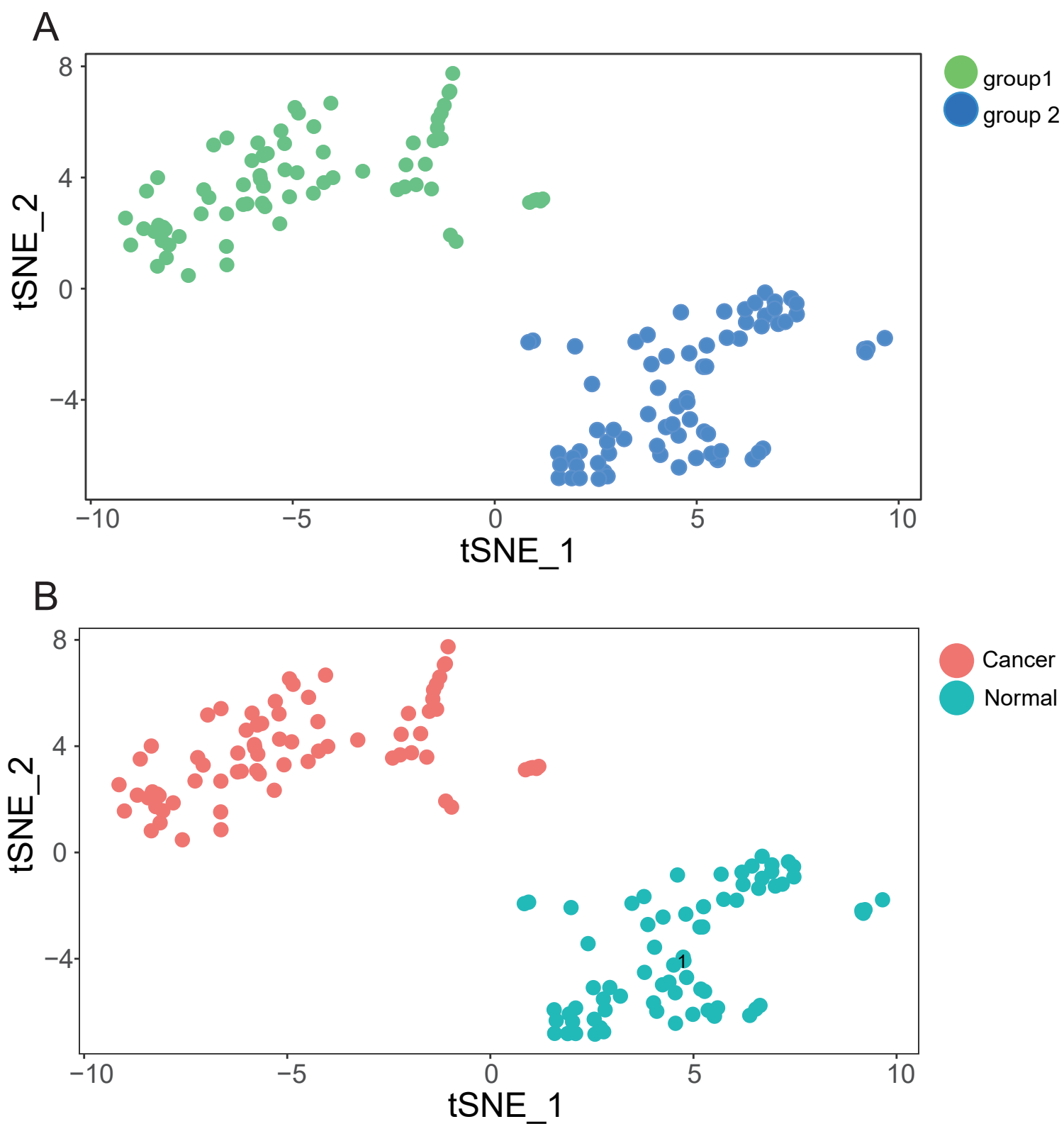

Supplement: Supplementary file 3 — Additional file 3: Figure S1. ESCC samples were clustered into two distinct tissues group by circRNA. [file 12935_2021_1852_MOESM3_ESM.pdf]

# Figure S4

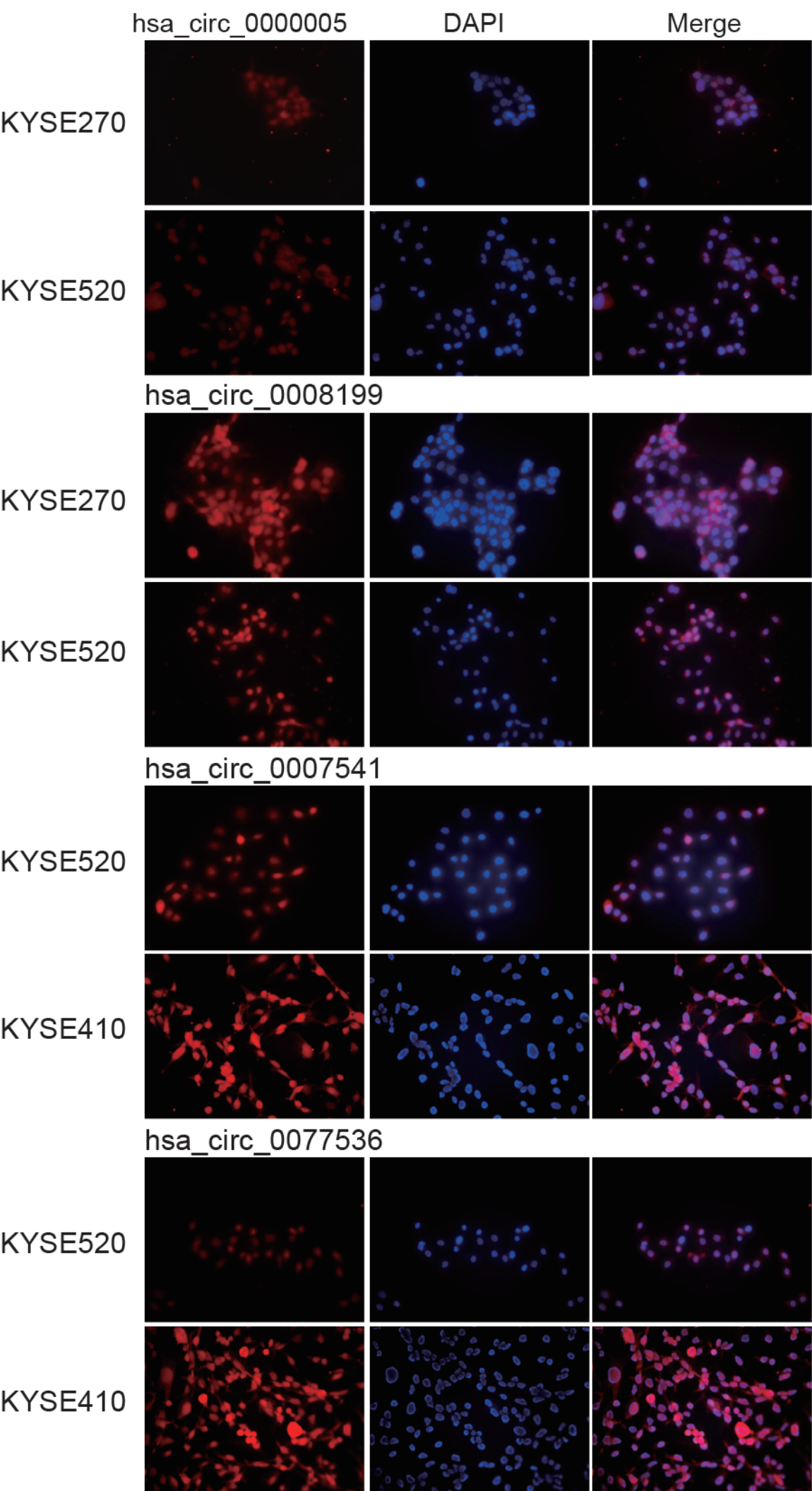

Supplement: Supplementary file 8 — Additional file 8: Figure S4. The subcellular localization experiment showed that all four circRNAs were located in the nucleus. [file 12935_2021_1852_MOESM8_ESM.pdf]
