# Supplementary material for: RNA sequencing reveals the expression profiles of circRNA and identifies a four-circRNA signature acts as a prognostic marker in esophageal squamous cell carcinoma
Source: Cancer Cell Int. 2021 Mar 4;21:151. doi: 10.1186/s12935-021-01852-9 (PMC7934454; doi:10.1186/s12935-021-01852-9)
Supplement: Supplementary file 4 — Additional file 4: Figure S2. Sanger sequencing results of the ten differentially expressed circRNAs. [file 12935_2021_1852_MOESM4_ESM.pdf]

Forward Primer
TGACAATCAACGAGGACAT

hsa_circ_0000099

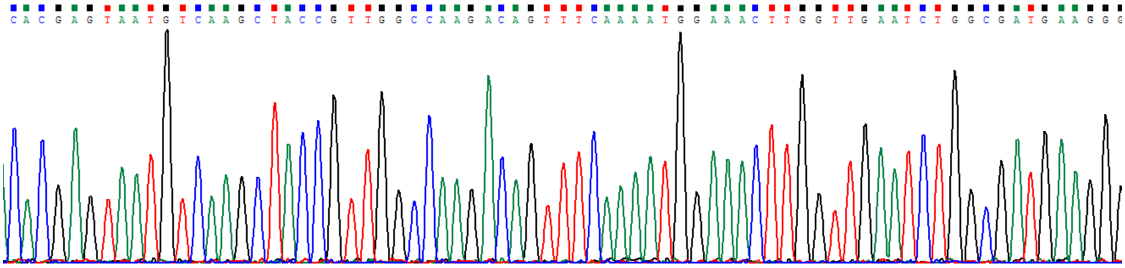

Reverse Primer
CATCGCCAGATTCAACCA

Forward Primer
AGAGCATCAGCAATACACAAGT

hsa_circ_0000479

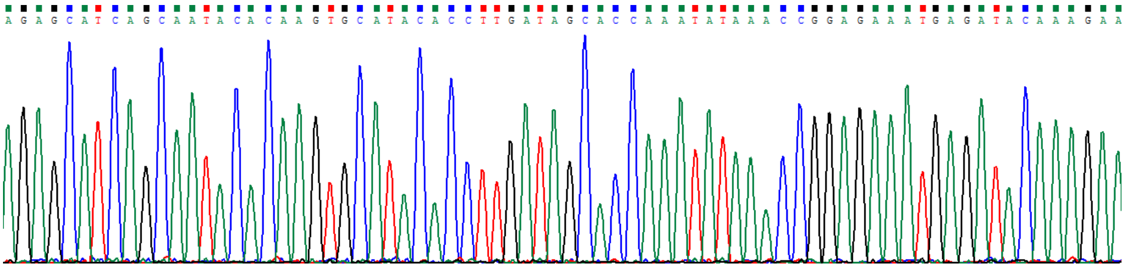

Reverse Primer
TTCTGACTGGCTTCCACCTA

Forward Primer
GAAGAATGAATAGAACAGAGGC

hsa_circ_0001707

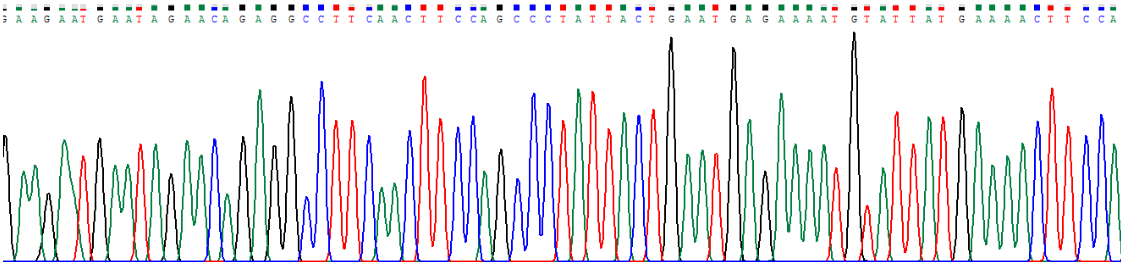

Reverse Primer
AGGCGAGAGTACATCTGG

Forward Primer
GATCTCTGCCAACTTCCTT

hsa_circ_0001821

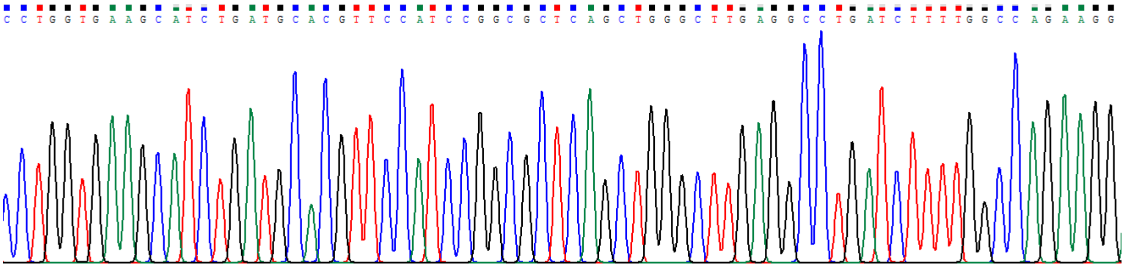

Reverse Primer

TTCCACCAGCGTTATTCC

Forward Primer

TGGATCACTAAGGTCTGTTCCC

hsa_circ_0001944

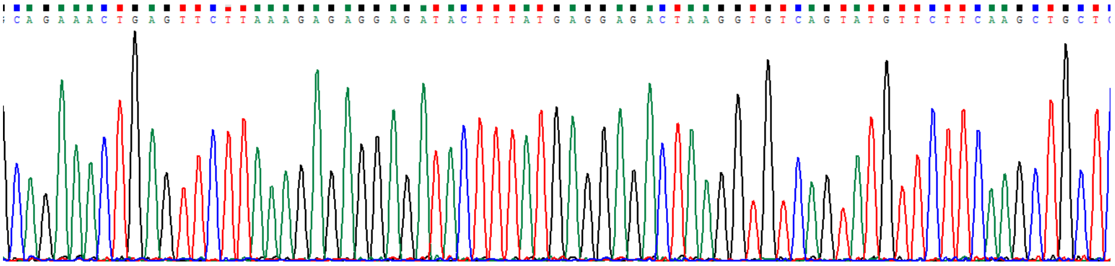

Reverse Primer

AGGAGCAGAGCAGCTTGAAGAA

Forward Primer

TGTCAGGGTCACATACTTTA

hsa_circ_0002874

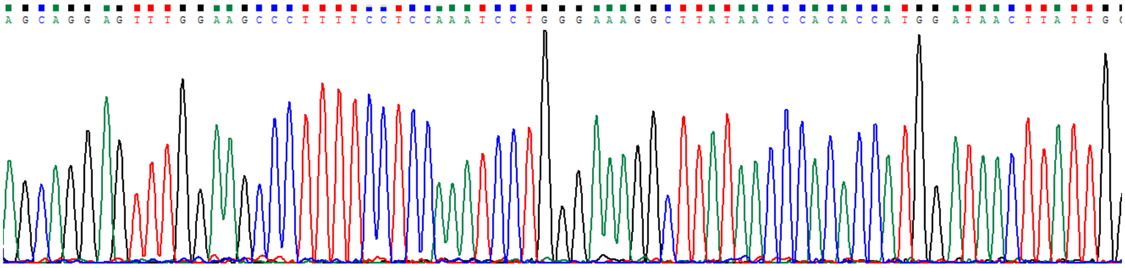

Reverse Primer

ATGCTGCATGATCTTCCA

Forward Primer

CTGTCACCATCTCCAAGC

hsa_circ_0003949

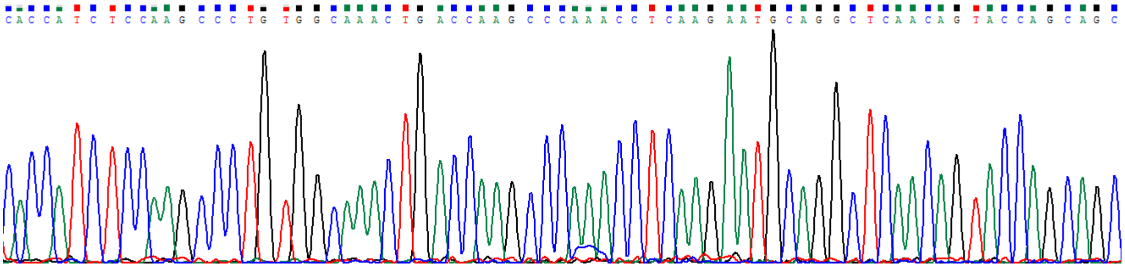

Reverse Primer

ACTCCACTGCCATTCTCC

Forward Primer

TCAACACGATAGCAGGAC

hsa_circ_0006867

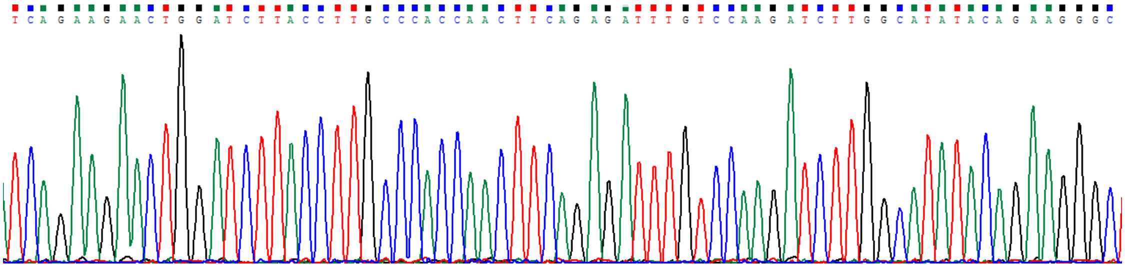

Reverse Primer
TGAACATCACAGCAACTCT

Forward Primer
GAGAAACAGACTCAGCAG

hsa_circ_0018064

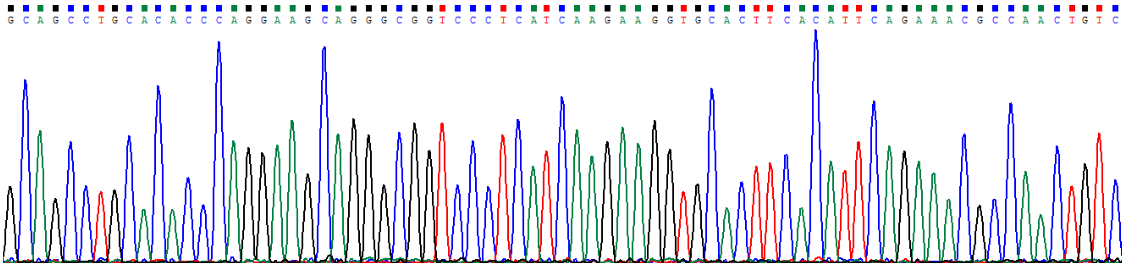

Reverse Primer
CCTCTTCAGTGGTGATGG

Forward Primer
GATAGCCACTACCTCCTCT

hsa_circ_0026782

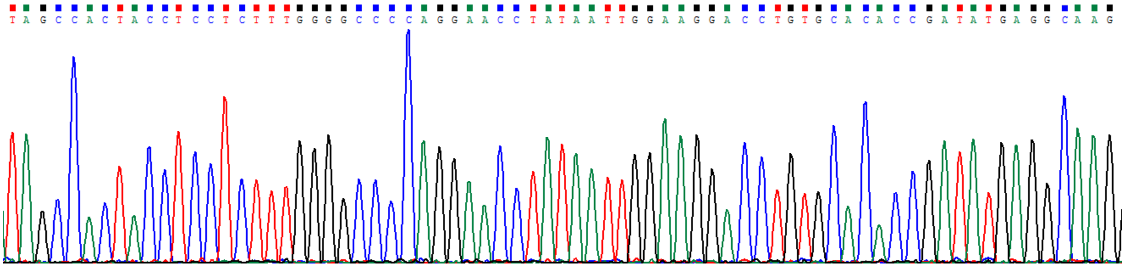

Reverse Primer
ACCATCCAACTCATCCCG
