# Supplementary material for: RNA sequencing reveals the expression profiles of circRNA and identifies a four-circRNA signature acts as a prognostic marker in esophageal squamous cell carcinoma
Source: Cancer Cell Int. 2021 Mar 4;21:151. doi: 10.1186/s12935-021-01852-9 (PMC7934454; doi:10.1186/s12935-021-01852-9)
Supplement: Supplementary file 6 — Additional file 6: Figure S3. Sanger sequencing results of the remaining 5 prognostic circRNAs except the 4 circRNAs in the signature. [file 12935_2021_1852_MOESM6_ESM.pdf]

Figure S3

Forward Primer
CAGAATGAAGACTTGAGGCGAATG

hsa_circ_0003028

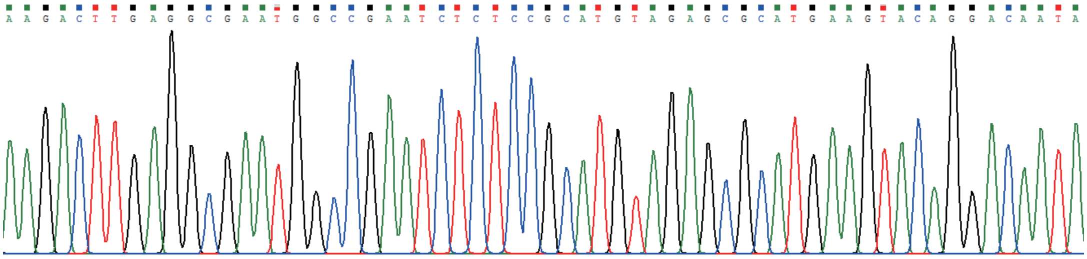

Reverse Primer
AAGAGATCCTCCTGGTGATATGTG

Forward Primer
CAACCTGAAGCAAATAAACCAACAC

hsa_circ_0007619

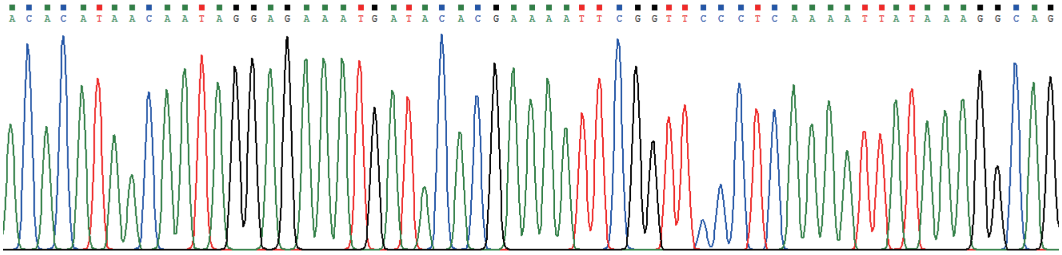

Reverse Primer
TGGCTGAGGACGCTCTGAA

Forward Primer
CTGTGGAAGCGAATAGCAGTAATAGC

hsa_circ_0002663

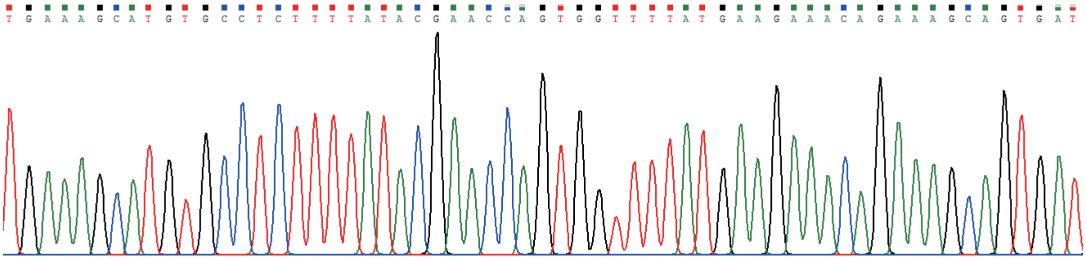

Reverse Primer
TTCTGATACTCTTGTCGTCTGTAGGC

Forward Primer
GAGTGAGGAATGAGGAAGTAGG

hsa_circ_0001726

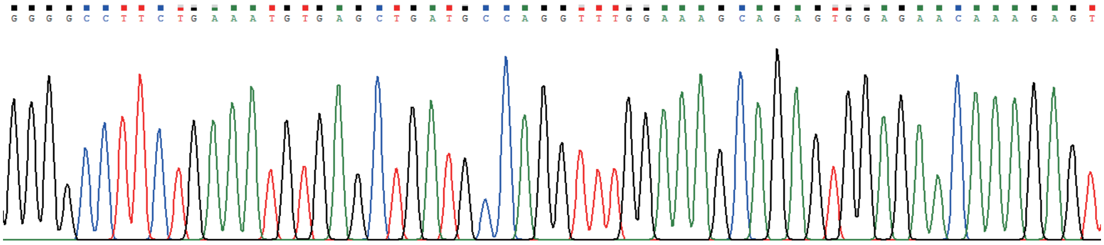

Reverse Primer
CCTGGAACGCTTCTTGTAGTT

Forward Primer
GCCTCCAGGATCACTAAGTTCAA

hsa_circ_0005314

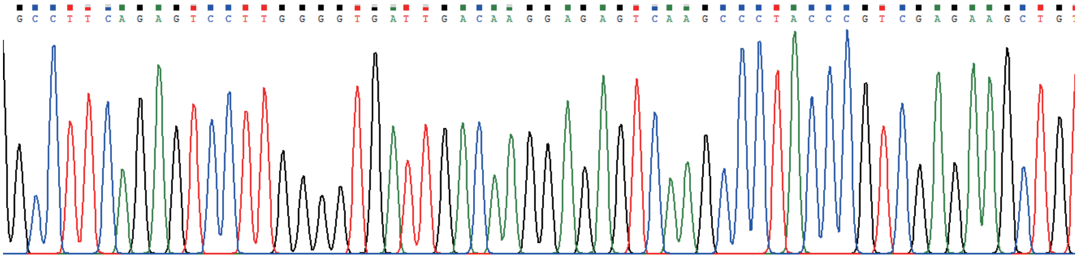

Reverse Primer
ACTCGGCTGTTCTTCTCTTCAC
