# Supplementary material for: RNA sequencing reveals the expression profiles of circRNA and identifies a four-circRNA signature acts as a prognostic marker in esophageal squamous cell carcinoma
Source: Cancer Cell Int. 2021 Mar 4;21:151. doi: 10.1186/s12935-021-01852-9 (PMC7934454; doi:10.1186/s12935-021-01852-9)
Supplement: Supplementary file 9 — Additional file 9: Table S5. Association of the circRNA signature with clinicopathological characteristics in ESCC patients. [file 12935_2021_1852_MOESM9_ESM.doc]

| **Supplementary table S5. Association of the circRNA signature with clinicopathological characteristics in ESCC patients** | | | | | | |
| --- | --- | --- | --- | --- | --- | --- |
| **Variables** | **Training group(n=73)** | | ***P*** | **Test group(n=125)** | | ***P*** |
|  | **Low risk *** | **High risk *** |  | **Low risk *** | **High risk *** |  |
| **Sex** |  |  | 0.990 |  |  | 0.795 |
| Female | 15 | 14 |  | 36 | 33 |  |
| Male | 22 | 22 |  | 27 | 29 |  |
| **Age** |  |  | 0.895 |  |  | 0.324 |
| ≤ 62 | 20 | 21 |  | 22 | 28 |  |
| > 62 | 17 | 15 |  | 41 | 34 |  |
| **Smoking** |  |  | 0.637 |  |  | 0.639 |
| No | 20 | 17 |  | 35 | 38 |  |
| Yes | 16 | 19 |  | 28 | 24 |  |
| **Drink** |  |  | 0.238 |  |  | 0.948 |
| **No** | 22 | 16 |  | 42 | 40 |  |
| **Yes** | 14 | 20 |  | 21 | 22 |  |
| **T stage** |  |  | 0.197 |  |  | 0.280 |
| T1, T2 | 12 | 6 |  | 23 | 16 |  |
| T3, T4 | 25 | 30 |  | 40 | 46 |  |
| **N stage** |  |  | 0.078 |  |  | 0.236 |
| **N0** | 22 | 13 |  | 44 | 36 |  |
| **N1-3** | 15 | 23 |  | 19 | 26 |  |
| **pTNM stage** |  |  | 0.049 |  |  | 0.233 |
| Stage Ⅱ | 22 | 13 |  | 46 | 37 |  |
| Stage Ⅲ | 15 | 20 |  | 16 | 22 |  |
| Stage Ⅳ | 0 | 3 |  | 1 | 3 |  |
| * Low risk ≤Median of risk score, High risk >Median of risk score; The Chi-squared test; P value <0.05 was considered significant. | | | | | | |
